# Three-dimensional comparison of intramuscular fat content between young and old adults

**DOI:** 10.1186/s12880-017-0185-9

**Published:** 2017-02-10

**Authors:** Akito Yoshiko, Maya Hioki, Nana Kanehira, Kiyoshi Shimaoka, Teruhiko Koike, Hisataka Sakakibara, Yoshiharu Oshida, Hiroshi Akima

**Affiliations:** 1 Graduate School of Medicine, Nagoya University, Nagoya, Japan; 2 Faculty of Human Wellness and Nutrition, Tokaigakuen University, Nagoya, Japan; 3 Faculty of Human Wellness, Tokaigakuen University, Miyoshi, Japan; 4 Research Center of Health, Physical Fitness & Sports, Nagoya University, Nagoya, Japan; 5 Graduate School of Education and Human Development, Nagoya University, Nagoya, Japan

**Keywords:** Intramuscular fat, Distribution characterization, Thigh muscles, Young adults, Old adults

## Abstract

**Background:**

Fat infiltration within skeletal muscle is known as intramuscular fat (IMF), which increases with aging. Studies have assessed IMF content, using the mid-thigh as a representative location. However, three-dimensional IMF distribution is not well understood. The aim of this study was to compare the IMF content in young and old adults by assessing its distribution along the length of the thigh.

**Methods:**

Consecutive transaxial images of the right thighs in 15 young (age, 21.0 ± 0.4) and 15 old (age, 70.7 ± 3.8) were obtained by magnetic resonance imaging. We measured IMF cross-sectional area (CSA), skeletal muscle CSA and calculated volume- and CSA-based IMF content for the quadriceps femoris (QF), hamstring (HM) and adductor (AD). CSA-based calculations were performed at every 10% of femur length (Lf), with 0% Lf and 100% Lf indicating the proximal and distal ends of femur.

**Results:**

IMF CSAs along the length of the thigh were similar in both age groups. In contrast, skeletal muscle CSAs in all three muscle groups were significantly lower in old adults than in young adults (variation: −15.2 to −1.6 cm^2^, *P* < 0.05). Thus, in volume-based measurements, the older adults had higher IMF contents than the younger adults (9.5% to 14.3% vs. 4.8% to 8.6%, respectively; *P* < 0.05). However, such age-dependent differences were not observed at the mid-thigh in the QF and AD.

**Conclusion:**

The results demonstrated an age-related increase in IMF content—confirmed in areas of the thigh—primarily based on finding lower amounts of skeletal muscle mass in CSAs in the older adults.

## Background

The age-related decrease in skeletal muscle mass is known as sarcopenia [1]. It is well understood that adipose tissue in skeletal muscle—that is, intramuscular fat (IMF)—accumulates as the muscle mass decreases during the aging process [2–4]. IMF is defined as adipose tissue infiltrated within a given muscle [5, 6]. Importantly, several studies reported that increased IMF resulted in lower insulin sensitivity [7, 8]. IMF is, in addition, inversely associated with muscle strength and mobility function [6, 9, 10]. Such evidence suggests that increased IMF would eventually induce physical dysfunction and may result in type 2 diabetes. Thus, an accurate understanding of how IMF accumulates during aging is essential.

To assess IMF in the lower limb muscles, medical imaging studies with magnetic resonance images (MRI) and computed tomography (CT), using single and multiple images, have been performed [2, 3, 11, 12]. Using such methods, IMF content, representing the fraction of fat tissue within a given muscle, was found to increase with age due to decreased skeletal muscle cross-sectional area (CSA) and/or increased IMF CSA values [2, 3, 13]. Several studies, attempting to address such age-dependent differences, calculated IMF content using a single axial image obtained at a region of the mid-thigh [14–16]. It was reported that skeletal muscle CSA decreased, in particular, at the muscle belly during aging [17] or disuse [18]. Thus, evaluations using a single image may overestimate differences in IMF content between young and old adults. Furthermore, Hasson *et al*. [19] showed that the characteristics of IMF content distribution along the length of the calf is U-shaped, indicating that it is higher at the proximal and distal regions than in the muscle belly of the calf. This inconsistent distribution of IMF content might affect the assessment of age-related differences. Unfortunately, in the context of three-dimensional IMF distribution, little is known about the differences in IMF content along the length of the thigh relative to the aging process.

The purpose of this study was to characterize the distribution pattern of IMF along the length of the thighs by comparing, using serial images, IMF content in young and old adults for three muscle groups. We hypothesized that, as reported for the calf, IMF would accumulate in a region-specific manner in the thighs and the presence of age-related differences in IMF content near the muscle belly, caused by decreased skeletal muscle and increased IMF in old adults.

## Methods

### Subjects

Fifteen young adults (age, 21.0 ± 0.4 years; 8 men and 7 women; height, 157.2 ± 6.4 cm; body weight, 56.5 ± 8.2 kg; BMI, 22 ± 2 kg/m^2^) and 15 old adults (age, 70.7 ± 3.8 years; 7 men and 8 women; height, 167.2 ± 10.9 cm; body weight, 62.3 ± 10.8 kg; BMI, 23 ± 2 kg/m^2^) volunteered to participate in this study. All of subjects were free from serious diseases. Prior to the study, the procedures, purposes, risks, and benefits associated with the study were explained to the subjects, who then provided written consent to participate in the study. This study was approved by the institutional review board of the Nagoya University Graduate School of Medicine and was conducted in accordance with the guidelines of the Declaration of Helsinki.

### Magnetic resonance imaging

Subjects were assessed with a 3.0-T whole-body MRI scanner (MAGNETOM Verio, Siemens Healthcare Diagnostics K.K, Tokyo, Japan). They were placed in a supine position, and images of the thigh were acquired using a body coil. T1-weighted spin-echo transaxial images of the right thigh were collected from 4 cm above the greater trochanter to a point 48 cm distal, using the following parameters: repetition time = 604 msec, echo time = 11 msec, voxel resolution = 0.75 mm, optimized field of view (FOV) = 256 × 256 mm, slice thickness = 10 mm, and interslice gap = 0 mm.

### Analysis of thigh composition

**Fig. 1:**
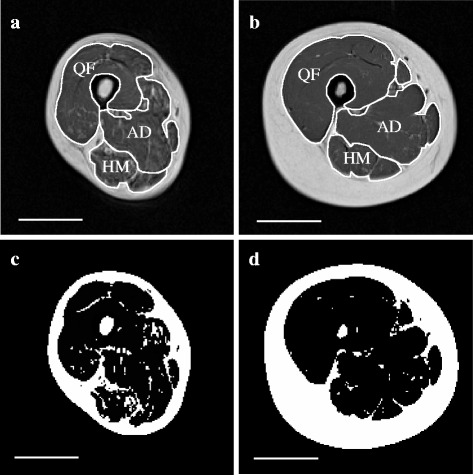
T1-weighted magnetic resonance images showing the three traced muscle groups, from women aged 71 years (**a**, **c**) and 21 years (**b**, **d**). (**c**) and (**d**) are binary images using thresholding technique. QF quadriceps femoris, HM hamstrings, AD adductor. Scale bar, 5 cm

Medical Image Processing, Analysis and Visualization software (version 4.4.0; National Institutes of Health, Bethesda, MD, USA) was used to analyze images on a personal computer (MacBook Pro, Apple Inc., Cupertino, CA, USA). The procedure for MRI data analysis was almost identical to a described previously method [15, 20]. Briefly, we assessed images from the greater trochanter to the lateral condyle for each subject to calculate muscle composition, as described below. Using these images, we identified the quadriceps femoris (QF), hamstring (HM), and adductor (AD) muscle groups in the whole thigh. Figure 1 shows representative MRIs of the mid-thigh of young and old adults.

In the first step of the analysis we used a well-established nonparametric nonuniform intensity normalization (N3) algorithm [20–22] to correct for image heterogeneity caused by suboptimal radiofrequency coil uniformity and gradient-driven eddy currents. This step was essential for subsequent analyses, which assumed homogeneous signal intensity across images. Optimized image correction parameters were determined (end tolerance 0.0001; maximum iterations 100; signal threshold 1; field distance 25 mm; subsampling factor 2; kernel full-width half-maximum 0.15; Wiener filter noise 0.01), and the same parameters were applied to all images from all subjects. The investigator then drew six regions of interest (ROIs) of 5 × 5 mm each. Three ROIs were placed on the vastus intermedius, and the other three ROIs were placed on subcutaneous adipose tissue. The vastus intermedius contains 99% of skeletal muscle [20] and was chosen so that we would obtain a pure skeletal muscle peak in the pixel number–signal intensity histogram. The total number of pixels within the six ROIs and a frequency distribution and histogram of all pixels and signal intensities were produced.

We measured IMF CSA, skeletal muscle CSA, and calculated IMF content of serial axial images, as described previously [20]. To ensure minimal investigator bias in the separation of skeletal muscle and adipose tissue in the pixel number–signal intensity histogram, we used the Otsu threshold method, a reliable histogram shape–based thresholding technique used in medical image analysis [23]. To minimize manual tracing–induced errors on thresholding values, the mean of five trials was used, and the values were applied to all muscles of interest. A binary image is shown in Fig. 1 (c) and (d). After carefully tracing the edge of each muscle group, the following parameters were calculated: 1) the total number of pixels within the muscle, 2) the number of pixels with a signal intensity lower than the threshold value (skeletal muscle pixel numbers), and 3) the number of pixels with a value higher than the threshold value (IMF pixel numbers). The proportion of IMF (i.e., IMF content) in each muscle was then calculated by using the following equation:

$$ \mathrm{I}\mathrm{M}\mathrm{F}\ \mathrm{content}\ \left(\%\right) = \left(\mathrm{IMF}\ \mathrm{pixel}\ \mathrm{numbers}\right)/\left[\left(\mathrm{skeletal}\ \mathrm{muscle}\ \mathrm{pixel}\ \mathrm{numbers}\right) + \left(\mathrm{IMF}\ \mathrm{pixel}\ \mathrm{numbers}\right)\right] \times 100 $$

Subsequently, the skeletal muscle CSA and IMF CSA were calculated by using the following equations:

$$ \mathrm{I}\mathrm{M}\mathrm{F}\ \mathrm{C}\mathrm{S}\mathrm{A}\ \left(\mathrm{c}{\mathrm{m}}^2\right) = \left(\mathrm{IMF}\ \mathrm{pixel}\ \mathrm{number}\right) \times {\left(\mathrm{FOV}/\mathrm{matrix}\ \mathrm{size}\right)}^2 $$

$$ \mathrm{Skeletal}\ \mathrm{muscle}\ \mathrm{C}\mathrm{S}\mathrm{A}\ \left(\mathrm{c}{\mathrm{m}}^2\right) = \left(\mathrm{skeletal}\ \mathrm{muscle}\ \mathrm{pixel}\ \mathrm{number}\right) \times {\left(\mathrm{FOV}/\mathrm{matrix}\ \mathrm{size}\right)}^2 $$

Finally, skeletal muscle volume and IMF volume were determined by using the following equations:

$$ \mathrm{I}\mathrm{M}\mathrm{F}\ \mathrm{volume}\ \left(\mathrm{c}{\mathrm{m}}^3\right) = \mathrm{M}\mathrm{ultiplying}\ \mathrm{the}\ \mathrm{sum}\ \mathrm{of}\ \mathrm{I}\mathrm{M}\mathrm{F}\ \mathrm{C}\mathrm{S}\mathrm{A} \times \left[\mathrm{slice}\ \mathrm{thickness}\ \left(1\ \mathrm{cm}\right) + \mathrm{slice}\ \mathrm{gap}\ \left(0\ \mathrm{cm}\right)\right] $$

$$ \mathrm{Skeletal}\ \mathrm{muscle}\ \mathrm{volume}\ \left(\mathrm{c}{\mathrm{m}}^3\right) = \mathrm{Multiplying}\ \mathrm{the}\ \mathrm{sum}\ \mathrm{of}\ \mathrm{skeletal}\ \mathrm{muscle}\ \mathrm{C}\mathrm{S}\mathrm{A} \times \left[\mathrm{slice}\ \mathrm{thickness}\ \left(1\ \mathrm{cm}\right) + \mathrm{slice}\ \mathrm{gap}\ \left(0\ \mathrm{cm}\right)\right] $$

$$ \mathrm{Volume}\hbox{-} \mathrm{based}\ \mathrm{I}\mathrm{M}\mathrm{F}\ \mathrm{content}\ \left(\%\right) = \mathrm{I}\mathrm{M}\mathrm{F}\ \mathrm{volume}/\left(\mathrm{IMF}\ \mathrm{volume} + \mathrm{skeletal}\ \mathrm{muscle}\ \mathrm{volume}\right) \times 100 $$

Body mass index (BMI; weight in kilograms divided by the square of the height in meters) did not significantly differ between the young and old adults; however, height was significantly shorter for old adults than for young adults. We expected that this would affect absolute values for IMF and skeletal muscle; thus, these parameters were normalized by body weight, when appropriate. Furthermore, IMF CSA and skeletal muscle CSA were expressed as values relative to the length of the femur (Lf). Images corresponding to the greater trochanter were expressed as 0% Lf, and those corresponding to the lateral condyle were expressed as 100% Lf. We calculated IMF CSA, skeletal muscle CSA and IMF content in 10% each along the Lf because we confirmed the age-related difference and the local accumulation.

All images were read in random order, and one investigator (AY) performed all image analyses. Test–retest reliability of IMF content was reported elsewhere [20]. Briefly, the intraclass correlation coefficient (ICC, 2.1) in individual muscles at the mid-thigh for 10 subjects ranged from 0.97 to 1.00 (all *P* < 0.001). The standard error of the measurement for the mid-thigh IMF content ranged from 2.9% (QF) to 4.6% (HM). The minimum difference ranged from 7.9% (QF) to 10.2% (AD).

### Statistical analysis

All values are expressed as mean ± standard deviation. The Student unpaired *t*-test was used to analyze differences in IMF and skeletal muscle volume and volume-based IMF content between groups. IMF CSAs, skeletal muscle CSAs, and IMF contents along the length of the thigh were analyzed using two-way (age × location) analysis of variance. In the case of two-factor interaction or a main effect, the Bonferroni post-hoc test was used to identify significant differences. The level of significance was set at *P* < 0.05. All statistical analysis was performed using IBM SPSS statistics (version 22.0; IBM, Tokyo, Japan).

## Results

### IMF volume, skeletal muscle volume, and IMF content in thigh muscle

Table 1 shows absolute and normalized IMF volumes, skeletal muscle volume, and volume-based IMF content in QF, HM, AD, and whole thigh. There was no significant difference between the young and old adults in IMF volume in any muscle group; however, skeletal muscle volume was significantly lower in the old adults than in the young adults. Normalized IMF volume of the HM was significantly higher in the old adults than in the young adults. Normalized skeletal muscle volume was significantly lower in the old adults than in the young adults, for all muscle groups. Volume-based IMF content in the QF, HM, and whole thigh was significantly higher in the old adults than in the young adults.

**Table 1:** Intramuscular fat volume, skeletal muscle volume, and intramuscular fat content of thigh muscle groups in young and old adults

	Quadriceps		Hamstrings		Adductor		Whole thigh	
	Young	Old	Young	Old	Young	Old	Young	Old
IMF	64.4 (32.1)	66.5 (15.0)	41.1 (24.4)	51.7 (13.4)	55.8 (39.2)	46.7 (16.3)	161.3 (93.1)	164.9 (39.4)
Skeletal muscle	1304.2 (373.2)	824.3 (247.2)*	438.5 (132.3)	316.7 (80.5)*	655.2 (226.4)	475.8 (161.1)*	2397.9 (712.1)	1616.8 (469.0)*
Normalized IMF	1.0 (0.4)	1.2 (0.2)	0.6 (0.3)	0.9 (0.2)*	0.9 (0.5)	0.8 (0.3)	2.5 (1.1)	2.9 (0.6)
Normalized skeletal muscle	20.7 (3.8)	14.4 (2.8)*	7.0 (1.5)	5.6 (1.1)*	10.4 (2.7)	8.2 (1.9)*	38.1 (7.6)	28.2 (5.2)*
Volume-based IMF content	4.8 (2.2)	7.8 (2.4)*	8.6 (4.7)	14.4 (3.7)*	8.3 (5.4)	9.5 (3.7)	6.5 (3.3)	9.7 (2.9)*

All values are mean (SD). IMF; intramuscular fat. Unit of IMF and skeletal muscle is cubic centimeter (cm^3^), normalized IMF and skeletal muscle is cubic centimeter per body weight (cm^3^/body weight), and volume-based.IMF content is percentage (%). The whole thigh comprises the QF, HM, and AD

Significant difference with young *P < 0.05

### IMF CSAs, skeletal muscle CSAs and IMF content for all thigh muscle groups

**Fig. 2:**
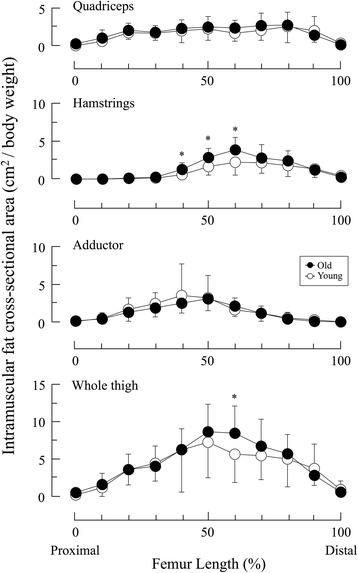
Cross-sectional area of intramuscular fat along the length of the femur in young and old adults. * *P* < 0.05 vs. young

**Fig. 3:**
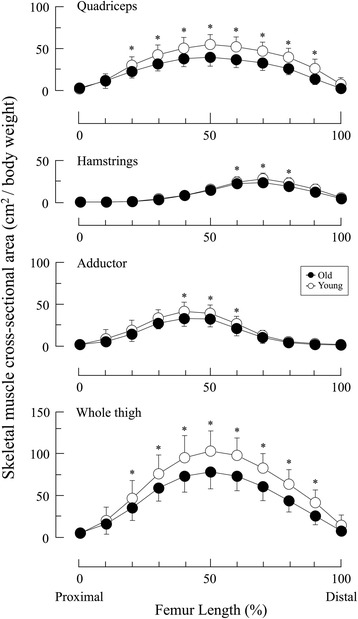
Cross-sectional area of skeletal muscle along the length of the femur in young and old adults. * *P* < 0.05 vs. old

The age-by-location interactions were found in the IMF CSAs for the HM and skeletal muscle CSAs for the QF and whole thigh. The IMF CSAs for the HM at 40% Lf to 60% Lf and the whole thigh at 60% Lf were significantly higher in the old adults than in the young adults (Fig. 2). The skeletal muscle CSAs for the QF and whole thigh was significantly lower in the old adults than in the young adults at almost all locations (20% Lf to 90% Lf). However, the skeletal muscle CSAs for the HM and AD were significantly lower in the old adults than in the young adults at three locations (HM: 60% Lf to 80% Lf; AD: 40% Lf to 60% Lf) (Fig. 3).

**Fig. 4:**
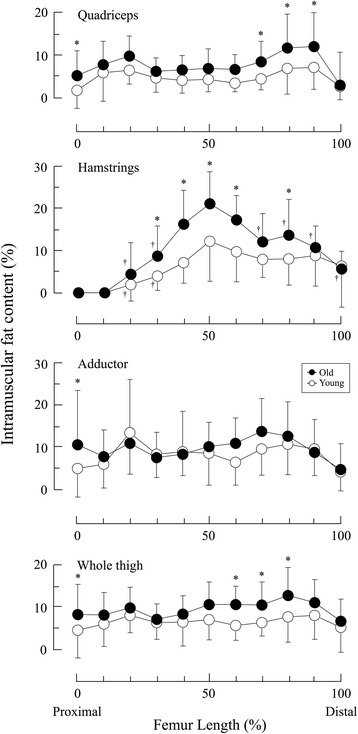
Distribution characterization of intramuscular fat content along the length of the femur in young and old adults. * *P* < 0.05 vs. young; † *P* < 0.05 vs. 50% of the femur length

We found age-by-location interaction of IMF content in the HM. IMF content, by CSA, was significantly higher for the old adults than for the young adults at 0% Lf and 70% Lf to 90% Lf in QF, at 30% Lf to 60% Lf and 80% Lf in HM, at 0% Lf in AD, and at 0% Lf and 60% Lf to 80% Lf in the whole thigh (Fig. 4).

### Comparison of IMF content at the mid-thigh and other regions

To investigate the validity of IMF content at the mid-thigh as a representative measure, we compared IMF content at the mid-thigh with that at other regions. HM was the only muscle for which there was a significant difference in IMF content between the mid-thigh and other regions. In the HM of the old adults, IMF content at the mid-thigh was significantly higher than that at 20% Lf, 30% Lf, and 70% Lf to 100% Lf. In the young adults, IMF content at the mid-thigh was significantly higher than that at 20% Lf and 30% Lf (Fig. 4).

## Discussion

This study compared two- and three-dimensional characteristics of IMF distribution (CSAs and volumes, respectively) in young and old adults. IMF CSAs were similar in the two age groups at almost all locations along the length of the thigh. However, skeletal muscle CSAs in all three muscle groups were significantly lower in old compared with young adults. Furthermore, there were significantly larger IMF contents at several locations along the length of the thigh for the QF and HM muscle groups and in one location in the AD muscle. These results suggested that age-related increases in IMF content in specific regions of the thigh were primarily caused by smaller skeletal muscle CSAs in old adults (sarcopenia), not by an increase in IMF CSAs.

### IMF CSA and skeletal muscle CSA comparisons in young and old adults

To compare young and old adults, the relative IMF CSA or volume within a given region of interest, expressed as IMF content, was used in previous studies [3, 9, 15, 19]. However, because this index is greatly affected by both IMF CSAs and skeletal muscle CSAs, we examined the IMF CSAs and skeletal muscle CSAs to determine their contributions to the total IMF content. As shown in Fig. 2, the distribution patterns of normalized IMF CSAs were similar in young and old adults. This seemed to contradict the hypothesis that IMF CSA would increase with age. However, skeletal muscle CSAs were significantly more diminished in old adults than in young adults, particularly in the muscle belly (Fig. 3). This was especially apparent in the QF, which clearly indicated the presence of sarcopenia. A similar pattern of muscle atrophy was also reported previously in the human QF with aging [17]. Thus, the atrophic pattern of skeletal muscle CSA we observed in old adults was as expected.

### IMF contents compared in young and old adults

IMF content may be calculated using IMF CSA and skeletal muscle CSA as previously described [3, 9, 15, 19]. In our study, the IMF content of the whole thigh, measured at the mid-thigh, was 6.9% ± 4.6% in young and 10.5% ± 5.2% in old adults (Fig. 4). Similar values were reported in previous studies [14, 24], however, we did not observe age-related differences for this parameter, contradicting previous results [15, 24]. The higher IMF content of the whole thigh in old adults compared in young adults was found at the length of the thigh from 60% to 80% Lf, a location slightly distal to the mid-thigh (Fig. 4). This specific regional difference was attributable to higher IMF CSAs and lower skeletal muscle CSAs in the QF and HM muscle groups of the old adults.

Because IMF content is inversely associated with skeletal muscle CSA [15], we hypothesized that IMF content in the old adults would be higher than in the young adults near the muscle belly in all muscle groups we tested. In the HM, the age-related differences were observed at several regions along the length of the thigh, including the muscle belly, leading to CSAs with higher levels of IMF and/or CSAs with lower levels of skeletal muscle in old than that in young adults (Figs. 2 to 4). In contrast, IMF CSAs were similar in both groups in the QF and AD although skeletal muscle CSAs were lower in the old adults. Therefore, in these regions, age-dependent changes in IMF CSAs, adding to the change of skeletal muscle CSAs, may affect the age-dependent changes in IMF content. Our results were inconsistent with those previously described by Overend *et al.* [2]. They reported that IMF CSA at the mid-thigh of the QF in old adults was approximately 1.5-fold higher than in younger adults. By normalizing IMF and skeletal muscle CSAs to body weight in our study, we minimized effects of physical characteristics. The discrepancies in these findings might have been caused by differences in imaging modalities, image analysis methodologies, or in ethnicities or lifestyles of the subjects.

### Muscle-specific characteristics of IMF content assessments of the thigh

For the HM only, we found that the IMF content was significantly higher in the mid-thigh than in the proximal and distal regions in both young and old adults, suggesting muscle-specific characteristics of IMF content assessments regardless of age (Fig. 4). Interestingly, these fluctuations were not great enough to affect the IMF content of the whole thigh. If we had used only a single image to quantify IMF content of HM, such as at the mid-thigh, as in previous studies [2, 6, 13], we would have overestimated this parameter in both young and old adults because the IMF contents at the mid-thigh HM were significantly higher than those at the proximal and distal regions (Fig. 4). In contrast, the distribution was constant in the QF, AD, and whole thigh along the length of the thigh. Our data showed that IMF CSAs and skeletal muscle CSAs (elements of the IMF content calculations) gradually increased from the muscle origin toward the belly and then gradually decreased toward the muscle insertion, in the same proportions.

Another interesting result of our study was that the distribution of IMF content assessments in the thigh was different from those reported in the calf. Hasson *et al*. [19] described IMF contents of the plantar flexor and dorsiflexor muscles that gradually increased toward the distal end of the lower limb. This was completely different from our observations in the thigh (Fig. 4). Possible explanations for this discrepancy include fiber type, architecture or muscle contribution during daily activities. IMF content calculated by MRI primarily reflects extramyocellular lipid (EMCL), not intramyocellular lipid (IMCL), in human thigh muscles and this would be associated with a relatively larger amount of fat deposition between muscle cells [16]. The amount of EMCL and IMCL did not differ in the soleus muscle in adolescents [25], but EMCL was 4.3 to 10.0 fold higher than IMCL in human thigh muscles [16]. Thus, accumulation patterns of EMCL may differ between muscle belly and both edges of muscle in young and old adults. However, the physiological reasons for region-specific IMF accumulation, including differences between thigh and calf muscles, remain unknown.

### Implication for accurate IMF content measurements using a single slice image

The volume-based IMF content in QF, HM and whole thigh in old adults was significantly higher than in the young group. However, we found no differences in IMF content in these groups based on a single slice image analysis in the QF and whole thigh (Table 1 and Fig. 4). This finding indicated that IMF content determined by single slice image analysis could provide misleading information about the age-related fat depot. Potential reasons for this would be region-specific changes in IMF CSAs and/or skeletal muscle CSAs (Figs. 2 and 3). Because IMF content represents the fat fraction within a given region of interest, increased IMF CSA and decreased skeletal muscle CSA could both greatly increase IMF content [15, 24]. Based on our results, it is important to track skeletal muscle size when measuring age-related changes of IMF content in thigh muscles.

## Conclusion

Age-related differences in IMF content were demonstrated in certain regions in the QF and HM and in the whole thigh. We found that age-related increases in IMF content exist in specific regions along the thigh. Furthermore, these region-specific differences might be determined by muscle atrophy, rather than by increased IMF quantity. By measuring characteristic of IMF content distribution along the thigh, assessment of this difference should be considered in evaluating location, especially in the HM muscle group.

## Abbreviations

AD: Adductor
CSA: Cross-sectional area
CT: Computed tomography
EMCL: Extramyocellular lipid
FOV: Field of view
HM: Hamstrings
IMAT: Intermuscular adipose tissue
IMCL: Intramyocellular lipid
IMF: Intramuscular fat
Lf: Length of femur
MRI: Magnetic resonance imaging
QF: Quadriceps femoris
ROI: Region of interest
